# Supplementary material for: Sex-steroid hormones and risk of postmenopausal estrogen receptor-positive breast cancer: a case–cohort analysis
Source: Cancer Causes Control. 2024 Feb 16;35(6):921–33. doi: 10.1007/s10552-024-01856-6 (PMC11130059; doi:10.1007/s10552-024-01856-6)
Supplement: Supplementary file 1 — Supplementary file1 (PDF 959 KB) [file 10552_2024_1856_MOESM1_ESM.pdf]

Online Supplementary Information

Sex-steroid hormones and risk of postmenopausal estrogen receptor-positive breast cancer: a case-cohort analysis

Frances EM Albers, Makayla WC Lou, S. Ghazaleh Dashti, Christopher TV Swain, Sabina Rinaldi, Vivian Viallon, Amalia Karahalios, Kristy A Brown, Marc J Gunter, Roger L Milne, Dallas R English, Brigid M Lynch

Journal: Cancer Causes and Control

Corresponding author: Brigid M Lynch[1,2,3] Brigid.Lynch@cancervic.org.au

1 Centre for Epidemiology and Biostatistics, Melbourne School of Population and Global Health, University of Melbourne, Melbourne, Australia

2 Cancer Epidemiology Division, Cancer Council Victoria, Melbourne, Australia

3 Physical Activity Laboratory, Baker Heart and Diabetes Institute, Melbourne, Australia.

Supplementary Contents

Online Resource 1: Characteristics of the subcohort and all eligible women for the case-cohort study within the Melbourne Collaborative Cohort Study 3

Online Resource 2: Handling of competing risks 5

Online Resource 3: Measured biomarkers and methods of measurement 7

Online Resource 4: Assessment of reliability of biomarker measurements 8

Online Resource 5: Reference standards for estradiol and testosterone 12

Online Resource 6: Normalization of biomarkers 14

Online Resource 7: Identification, measurement and modelling of sociodemographic and lifestyle confounders 16

Online Resource 8: Identification and selection of biomarker confounders 18

Online Resource 9: Characteristics of the case-cohort after *post-hoc* exclusions 20

Online Resource 10: Sensitivity analyses excluding estrogen receptor-negative/progesterone receptor-positive tumors and tumors of unknown hormone receptor status 23

Online Resource 11: Sensitivity analyses excluding cases and deaths that occurred within one year of blood draw at the second follow-up wave 25

Online Resource 12: Sensitivity analyses for naturally postmenopausal women with a recorded age at menopause 27

Online Resource 13: Quartile minimum, median and maximum values 29

Supplementary References 31

Online Resource 1: Characteristics of the subcohort and all eligible women for the case-cohort study within the Melbourne Collaborative Cohort Study

Online Resource Table 1.1 Characteristics of the subcohort and all eligible women

	**All Eligible** N = 10,669		**Subcohort** N = 999	
**Age at Blood Collection** (Years; Median, IQR)	68.0	(61.0, 74.0)	67.0	(61.0, 74.0)
**Dietary Calcium Intake** (mg/d; Median, IQR)	801.3	(602.9, 1044.2)	816.9	(613.4, 1045.3)
**Total Carotenoid Intake from Diet** (mcg/d; Median, IQR)	18093	(13519, 23669)	17287	(13352, 23188)
**Southern European Migrant Status** (N, %)				
No	8376	78.5%	782	78.3%
Yes	2293	21.5%	217	21.7%
**Socioeconomic Disadvantage** (N, %)				
Quintile 1: Most Disadvantaged	1842	17.3%	155	15.5%
Quintile 2	2143	20.1%	201	20.1%
Quintile 3	1578	14.8%	151	15.1%
Quintile 4	2056	19.3%	194	19.4%
Quintile 5: Least Disadvantaged	3042	28.5%	297	29.8%
**Education** (N, %)				
Primary School or Some High / Technical School	6730	63.1%	650	65.1%
Completed High / Technical School	1910	17.9%	151	15.1%
Completed Tertiary Degree / Diploma	2029	19.0%	198	19.8%
**Smoking Status** (N, %)				
Never Smoked	7667	71.9%	735	73.6%
Ever Smoked	3002	28.1%	264	26.4%
**Lifetime Alcohol Consumption** (N, %)				
Life Abstention	4117	38.7%	374	37.4%
≤ 19 g/d	5815	54.7%	563	56.4%
20 to 29 g/d	434	4.1%	31	3.1%
30 to 39 g/d	148	1.4%	19	1.9%
≥ 40 g/d	111	1.0%	12	1.2%
**Body Mass Index** (N, %)				
Normal (≥ 18.5 to < 25 kg/m^2)	4412	41.4%	422	42.2%
Overweight (≥ 25 to < 30 kg/m^2)	3978	37.3%	358	35.8%
Obese (≥ 30 kg/m^2)	2279	21.4%	219	21.9%
**Physical Activity[a] (**N, %)				
Insufficiently Active	3223	32.6%	293	31.2%
Sufficiently Active	2598	26.3%	263	28.0%
Highly Active	4075	41.2%	383	40.8%

N: Number. IQR: Interquartile range. g/d: Grams per day. mg/d: Milligrams per day. mcg/d: Micrograms per day. kg/m^2: Kilograms per meters squared.

[a] Physical activity was measured as total weighted minutes of walking, moderate- and vigorous-intensity recreation- and transport-related physical activity (MVPA) per week at the second follow-up wave. Insufficiently active was defined as < 150 total weighted minutes of MVPA per week, sufficiently active was defined as 150 to ≤ 300 total weighted minutes of MVPA per week, and highly active was defined as > 300 total weighted minutes of MVPA per week.

Missing data for covariates include: 8 for socioeconomic disadvantage, 773 for physical activity and 44 for lifetime alcohol consumption.

Southern European Migrant status, socioeconomic disadvantage, education, smoking status, lifetime alcohol consumption, body mass index, dietary calcium intake and total carotenoid intake from diet were measured at baseline. Age at blood collection and physical activity were measured at the second follow-up wave. Age at menopause was not estimated for all eligible women and was thus not presented in the table.

Online Resource 2: Handling of competing risks

To minimize the impact of death as a competing risk and maximize the retention of eligible cases in this relatively older cohort, follow-up was chosen to end on participants' 86th birthday (as informed by Online Resource Table 2.1). Accommodating competing risks is acknowledged as a challenge in the causal inference literature, especially in the absence of time-varying data [1, 2].

Online Resource Table 2.1 Competing risk of death by age

**Attained Age**[a]	**Breast Cancer Cases**[b]			**Deaths from Other Causes (Subcohort Non-Failures)**[b]			
	**Incident**	**Cumulative**	**Cumulative Percent (%)**[c]	**Incident**	**Cumulative**	**Remaining Subcohort Non-Failures**	**Cumulative Risk (%)**[d]
**54**	0	0	0.0	0	0	932	0.0
**55**	1	1	0.2	0	0	932	0.0
**56**	2	3	0.7	0	0	932	0.0
**57**	2	5	1.1	0	0	932	0.0
**58**	3	8	1.8	1	1	931	0.1
**59**	4	12	2.7	0	1	931	0.1
**60**	8	20	4.6	1	2	930	0.2
**61**	3	23	5.3	0	2	930	0.2
**62**	2	25	5.7	0	2	930	0.2
**63**	9	34	7.8	0	2	930	0.2
**64**	8	42	9.6	2	4	928	0.4
**65**	8	50	11.4	0	4	928	0.4
**66**	9	59	13.5	2	6	926	0.6
**67**	19	78	17.8	4	10	922	1.1
**68**	9	87	19.9	0	10	922	1.1
**69**	25	112	25.6	0	10	922	1.1
**70**	19	131	30.0	3	13	919	1.4
**71**	22	153	35.0	3	16	916	1.7
**72**	19	172	39.4	5	21	911	2.3
**73**	16	188	43.0	2	23	909	2.5
**74**	20	208	47.6	3	26	906	2.9
**75**	16	224	51.3	4	30	902	3.3
**76**	17	241	55.1	4	34	898	3.8
**77**	23	264	60.4	8	42	890	4.7
**78**	16	280	64.1	9	51	881	5.8

**79**	17	297	68.0	6	57	875	6.5
**80**	15	312	71.4	9	66	866	7.6
**81**	18	330	75.5	12	78	854	9.1
**82**	9	339	77.6	15	93	839	11.1
**83**	18	357	81.7	11	104	828	12.6
**84**	15	372	85.1	13	117	815	14.4
**85**	13	385	88.1	8	125	807	15.5
**86**	12	397	90.8	13	138	794	17.4
**87**	9	406	92.9	14	152	780	19.5
**88**	9	415	95.0	15	167	765	21.8
**89**	7	422	96.6	16	183	749	24.4
**90**	5	427	97.7	9	192	740	25.9
**91**	3	430	98.4	8	200	732	27.3
**92**	2	432	98.9	12	212	720	29.4
**93**	3	435	99.5	6	218	714	30.5
**94**	0	435	99.5	3	221	711	31.1
**95**	0	435	99.5	2	223	709	31.5
**96**	1	436	99.8	2	225	707	31.8
**97**	1	437	100.0	0	225	707	31.8
**98**	0	437	100.0	1	226	706	32.0
**Total**	437	437	100.0	226	226	480	

[a] Attained age is floored age e.g., age 85 is age 85.0-85.9.

[b] Total number in the case-cohort after 32 women were retrospectively excluded due to estradiol values at or above 29.3 pg/mL (or 107.6 pmol/L), one woman was excluded due to non-participation in the second follow-up wave despite providing a blood sample, and four cases outside the subcohort were retrospectively disqualified (diagnosis by death certificate only or non-adenocarcinoma breast cancer).

[c] Calculated as the cumulative number of breast cancer cases by the attained age divided by the total number of breast cancer cases (N = 437), multiplied by 100.

[d] Calculated as the cumulative number of deaths from other causes by the attained age among subcohort non-failures divided by the remaining subcohort non-failures by the attained age, multiplied by 100.

Online Resource 3: Measured biomarkers and methods of measurement

All biomarkers were measured at the Nutrition and Metabolism Branch, International Agency for Research on Cancer (IARC) as outlined in Online Resource Table 3.1.

Online Resource Table 3.1 Biomarkers and methods of measurement

Biological Pathway	Measurement Method	Biomarker (Unit of Measurement)	Dispatch 1 LLOQ/ULOQ	Dispatch 2 LLOQ/ULOQ
Inflammation	Electrochemiluminescent methods (Meso Scale Discovery, Rockville, MD)	Adiponectin (pg/mL)	78,125/1.6 x 10^8	5,000/1.6 x 10^8
		Leptin (pg/mL)	68.5/100,000	68.5/100,000
		TNF-α (pg/mL)	0.33/696	0.34/696
		IFN-γ (pg/mL)	0.33/3160	0.39/3160
		IL-6 (pg/mL)	0.09/1486	0.18/1486
		IL-10 (pg/mL)	0.095/772	0.09/772
		IL-8 (pg/mL)	0.135/1204	0.07/1204
		CRP (pg/mL)	12,544/1.96 x 10^8	12,500/1.96 x 10^8
Insulin/IGF-signaling	Enzyme-linked immunosorbent assay by ALPCO (Salem, USA)	C-peptide (ng/mL)	0.31/14.3	0.31/14.8
	Immunoassay methods by R&D Systems (Biotechne, Minneapolis, USA)	IGF-1 (ng/mL)	6.3/400[a]	12.5/400
		IGFBP-3 (ng/mL)	78.1/5,000	78.5/5,000
	Electrochemiluminescent methods (Meso Scale Discovery, Rockville, MD)	Insulin (pg/mL)	34.5/50,000	34.5/50,000
Sex-steroid hormones and SHBG	Liquid chromatography-mass spectrometry system consisting of an ultra-high-performance liquid chromatograph (Agilent 1290, Agilent, Santa Clara, CA) and a QTRAP 5500 mass spectrometer (SCIEX, Framingham, MA)	Testosterone (pg/mL)	7.5/15,000	7.5/15,000
		Androstenedione (pg/mL)	7.5/15,000	7.5/15,000
		DHEA (pg/mL)	125/250,000	125/250,000
		Progesterone (pg/mL)	7.5/15,000	7.5/15,000
		Estrone (pg/mL)	1.25/2,500	1.25/2,500
		Estradiol (pg/mL)	1.25/2,500	1.25/2,500
	Solid-phase "sandwich" enzyme-linked immunoassay (DRG International, Springfield, NJ)	SHBG (nmol/L)	4/260	4/260

LLOQ: Lower limit of quantification. ULOQ: Upper limit of quantification. IGF: Insulin-like growth factor. TNF-α: Tumor necrosis factor-alpha. INF-γ: Interferon gamma; IL-6: Interleukin-6; IL-10: Interleukin-10. IL-8: Interleukin-8; CRP: C-reactive protein. C-peptide: Connecting peptide. IGF-1: Insulin-like growth factor-1. IGFBP-3: Insulin-like growth factor binding protein-3. DHEA: Dehydroepiandrosterone. SHBG: Sex hormone binding globulin. QTRAP: Triple Quad Linear Ion Trap. pg/mL: Picograms per milliliter. ng/mL: Nanograms per milliliter. nmol/L: Nanomoles per liter. pmol/L: Picomoles per liter.

[a] The ULOQ is 100ng/mL for IGF-1 measured in batch 5 only.

Online Resource 4: Assessment of reliability of biomarker measurements

Three quality control (QC) samples were created for the reliability study, one for each of three body mass index (BMI) categories at baseline: normal (≥ 18.5 kg/m^2 to < 25 kg/m^2, QC1), overweight (≥ 25 kg/m^2 to < 30 kg/m^2, QC2), and obese (≥ 30 kg/m^2, QC3). Each QC sample contained the pooled plasma of 38 women who met the initial eligibility criteria for the case-cohort study and had at least three vials of plasma remaining at the second follow-up wave (F2). Two replicates of each QC sample were used in each batch. Intra-assay and inter-assay coefficients of variation (CVs) were calculated for each quality control sample for each biomarker to assess the reliability of biomarker measurements within and across batches, respectively (Online Resource Table 4.1).

In addition, linear mixed-effects regression models with a fixed effect for dispatch and random crossed-effects for BMI and batch were specified for each biomarker. These models were used to estimate within-batch and between-batch intra-class correlation coefficients (ICCs) (Online Resource Table 4.2). The delta method was used to calculate 95% confidence intervals. The within-batch ICCs estimate the correlation between biomarker measurements within the same batch and BMI category, and the between-batch ICCs estimate the correlation between biomarker measurements within the same BMI category (but not the same batch), after correction for dispatch effects [3].

Online Resource Table 4.1 Overall intra-assay and inter-assay coefficients of variation for each biomarker

Biomarker	Overall Intra-Assay CV (%)			Overall Inter-Assay CV (%)		
	QC1	QC2	QC3	QC1	QC2	QC3
**Androstenedione**	3.00	2.66	2.33	5.63	5.41	4.95
**DHEA**	2.97	3.76	3.98	3.33	3.39	4.29
**Estradiol**	5.87	5.92	6.73	6.04	8.03	7.99
**Estrone**	3.07	4.17	3.60	7.82	6.28	6.43
**Progesterone**	4.08	4.73	4.73	10.79	13.73	12.28
**Testosterone**	2.64	2.74	2.93	5.15	5.42	4.99
**SHBG**	3.14	2.94	2.54	8.11	6.85	9.03
**IGF-1**	3.36	4.19	2.89	12.85	13.61	11.53
**IGFBP-3**	3.74	1.63	2.64	5.13	6.00	5.66
**IFN-γ**	9.55	4.82	2.94	10.95	9.64	10.85
**IL-10**	7.82	7.62	7.97	11.84	14.59	17.27[a]
**IL-6**	6.14	6.44	5.57	11.20	11.59	12.00
**IL-8**	6.07	5.51	4.82	11.04	11.26	9.88
**TNF-α**	4.68	3.71	3.72	34.11[b]	34.12[c]	33.02[d]
**Insulin**	5.66	3.18	4.00	9.18	9.00	8.36
**Leptin**	3.35	5.45	3.51	9.18	7.23	7.39
**Adiponectin**	4.01	6.73	2.82	24.55[e]	20.56[f]	16.48[g]
**CRP**	6.23	3.44	2.93	6.27	5.92	9.82
**C-Peptide**	10.23	1.94	10.11	4.30	4.75	5.51

CV: Coefficient of variation. QC1: Quality control for normal body mass index (≥ 18.5 kg/m^2 to < 25 kg/m^2). QC2: Quality control for overweight body mass index (≥ 25 kg/m^2 to < 30 kg/m^2). QC3: Quality control for obese body mass index (≥ 30 kg/m^2). DHEA: Dehydroepiandrosterone. SHBG: Sex hormone binding globulin. IGF-1: Insulin-like growth factor-1. IGFBP-3: Insulin-like growth factor binding protein-3. IFN-γ: Interferon gamma. IL-10: Interleukin-10. IL-6: Interleukin-6. IL-8: Interleukin-8. TNF-α: Tumor necrosis factor-alpha. CRP: C-reactive protein. C-peptide: Connecting peptide.

Dispatch-specific CVs are provided where overall inter-assay CVs are above 15%.

[a] CV for dispatch one: 13.44%; CV for dispatch two: 13.77%.

[b] CV for dispatch one: 12.11%; CV for dispatch two: 11.50%.

[c] CV for dispatch one: 12.84%; CV for dispatch two: 12.27%.

[d] CV for dispatch one: 11.06%; CV for dispatch two: 10.94%.

[e] CV for dispatch one: 15.06%; CV for dispatch two: 3.41%.

[f] CV for dispatch one: 11.46%; CV for dispatch two: 6.33%.

[g] CV for dispatch one: 8.12%; CV for dispatch two: 3.74%.

All calculated overall intra-assay CVs were below 10% except for QC1 (10.23%) and QC3 (10.11%) for C-peptide (Online Resource Table 4.1). All calculated overall inter-assay CVs were below 15% except for: QC2 (17.27%) for interleukin (IL)-10; QC1 (34.11%), QC2 (34.12%) and QC3 (33.02%) for tumor necrosis factor (TNF)-α; and QC1 (24.55%), QC2 (20.56%) and QC3 (16.48%) for adiponectin. Overall inter-assay CVs above 15% were likely

attributable to dispatch effects. Dispatch-specific CVs are provided, where applicable. Normalization of biomarker values before analyses accounted for dispatch effects (Online Resource 6).

Online Resource Table 4.2 Estimated intra-batch and inter-batch reliability intra-class correlation coefficients for each biomarker

Biomarker	Intra-Batch ICC % (95% CI)[a]	Inter-Batch ICC % (95% CI)[a]
**Androstenedione**	87 (71, 100)	76 (46, 100)
**DHEA**	69 (38, 99)	58 (18, 98)
**Estradiol**	97 (93, 100)	97 (92, 100)
**Estrone**	90 (75, 100)	88 (71, 100)
**Progesterone**	85 (68, 100)	69 (33, 100)
**Testosterone**	78 (52, 100)	73 (41, 100)
**SHBG**	96 (91, 100)	93 (83, 100)
**IGF-1**	69 (55, 84)	5 (0, 15)
**IGFBP-3**	60 (42, 77)	7 (0, 19)
**IFN-γ**	88 (75, 100)	71 (38, 100)
**IL-10**	90 (78, 100)	80 (53, 100)
**IL-6**	78 (62, 94)	39 (0, 79)
**IL-8**	99 (97, 100)	97 (92, 100)
**TNF-α**	89 (80, 97)	41 (0, 81)
**Insulin**	99 (96, 100)	96 (90, 100)
**Leptin**	99 (99, 100)	99 (97, 100)
**Adiponectin**	59 (26, 91)	46 (4, 87)
**CRP**	95 (87, 100)	91 (77, 100)
**C-Peptide**	90 (76, 100)	90 (76, 100)

ICC: Intra-class correlation coefficient. CI: Confidence interval. DHEA: Dehydroepiandrosterone. SHBG: Sex hormone binding globulin. IGF-1: Insulin-like growth factor-1. IGFBP-3: Insulin-like growth factor binding protein-3. IFN-γ: Interferon gamma. IL-10: Interleukin-10. IL-6: Interleukin-6. IL-8: Interleukin-8. TNF-α: Tumor necrosis factor-alpha. CRP: C-reactive protein. C-peptide: Connecting peptide.

[a] Lower bounds were truncated at 0% and upper bounds were truncated at 100%, where appropriate.

All estimated intra-batch ICCs were above 80% except for dehydroepiandrosterone (DHEA, 69%), testosterone (78%), insulin-like growth factor (IGF)-1 (69%), insulin-like growth factor binding protein (IGFBP)-3 (60%), interleukin (IL)-6 (78%) and adiponectin (59%) (Online Resource Table 4.2). All estimated inter-batch ICCs were above 70% except for

DHEA (58%), progesterone (69%), IGF-1 (5%), IGFBP-3 (7%), IL-6 (39%), TNF-α (41%) and adiponectin (46%). Low inter-batch ICCs were likely indicative of batch effects. Normalization of biomarker values before analyses accounted for batch effects (Online Resource 6).

The estimated 95% confidence intervals presented in Online Resource Table 4.2 were wide and/or required truncation. Constructing confidence intervals for ICCs is difficult when one or more factors has few levels (in this case, BMI) [4]. This may explain why estimated lower bounds and upper bounds could be less than 0% and exceed 100%, respectively. Bounds were truncated at 0% or 100% where appropriate.

Online Resource 5: Reference standards for estradiol and testosterone

Reference standards for estradiol and testosterone were used to evaluate assay performance at the Nutrition and Metabolism Branch, IARC. Reference standards with known plasma concentrations for estradiol and testosterone were obtained from the Clinical Standardization Programs at the US Centers for Disease Control and Prevention. For each biomarker, fifteen samples were chosen to be representative of the range of values for postmenopausal women. The reference samples were aliquoted into 60 samples of four replicates and measured in two batches. Two sets of replicates were measured in each batch using a liquid chromatography-mass spectrometry system. Assay details are described in the main text and Online Resource 3.

The concentrations of the reference standards and their corresponding measurements at IARC were highly correlated. The calculated Pearson correlation coefficients (also known as the validity coefficients) for estradiol and testosterone were 0.987 and 0.997, respectively. For estradiol, the measured values slightly overestimated the true values at higher concentrations (Online Resource Fig. 5.1). The measured values for testosterone were very close to the true values (Online Resource Fig. 5.2).

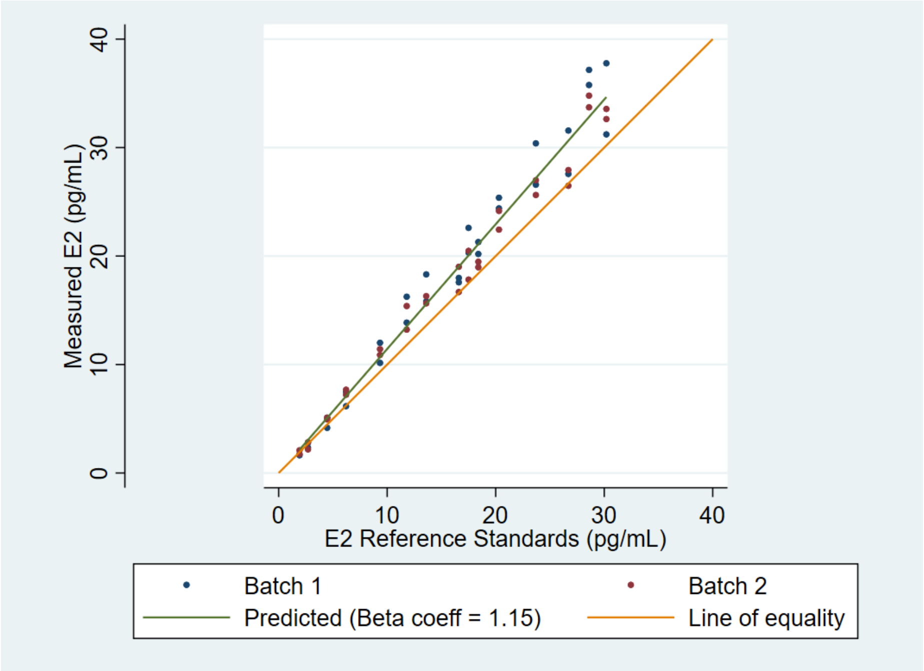

Online Resource Fig. 5.1 Correlation plot for estradiol

E2: Estradiol. The beta-coefficient for the regression line used to generate predicted values was 1.15.

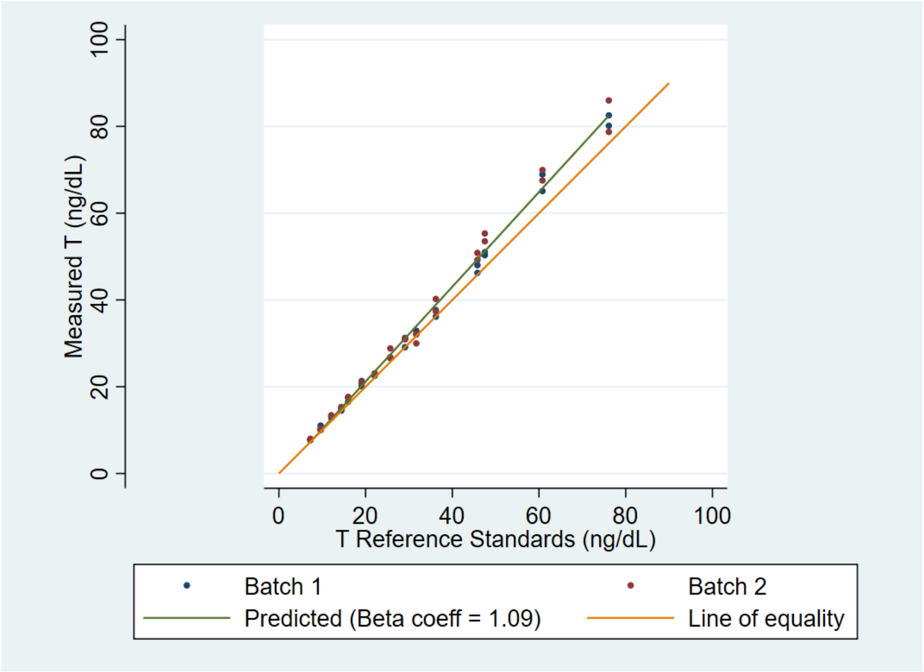

Online Resource Fig. 5.2 Correlation plot for testosterone

T: Testosterone. The beta-coefficient for the regression line used to generate predicted values was 1.09.

Online Resource 6: Normalization of biomarkers

To prepare biomarker data for normalization, values above the upper limit of quantification (ULOQ), or below the lower limit of quantification (LLOQ) or lower limit of detection (LOD), were first imputed as the ULOQ or LLOQ/2 respectively. Biomarker concentrations were converted to standard molar units where possible before log_2-transformation. Reasons for missing data were also identified and were mostly attributable to missing samples or technical issues pertaining to a specific biomarker measurement.

The linear mixed-effects models for normalization were specified for each biomarker to include a batch-specific random effect, and fixed effects for dispatch, time since last meal, and biological sources of variation. Time since last meal was centered at twelve hours and modelled using restricted cubic splines. Biological sources of variation were identified *a priori* and included case status, age at blood collection, BMI at F2, Southern European migrant status and smoking status at F2. Normalized values were estimated as the sum of the residual error, the regression term for the constant, and biological covariates [5]. This calculation retains biological sources of variation but removes nuisance variation by essentially 'fixing' the data as if every measurement came from the first dispatch (reference category) and all participants were fasting for T = 12 hours [5].

Prior to normalization, the linear mixed-effects models were used to estimate residual ICCs for the total proportion of variation attributable to batch (Online Resource Table 6.1).

Online Resource Table 6.1 Estimated residual intra-class correlation coefficients for the total proportion of variation attributable to batch for measured biomarkers

Biomarker	Residual ICC % (95% CI)
**Androstenedione**	0.47 (0.03, 6.70)
**DHEA**	1.02 $x10^{-13}$ (1.02 $x10^{-13}$, 1.02 $x10^{-13}$)
**Estradiol**	1.17 (0.28, 4.80)
**Estrone**	0.98 (0.20, 4.76)
**Progesterone**	2.29 (0.83, 6.20)
**Testosterone**	1.36 (0.36, 4.97)
**SHBG**	2.31 (0.85, 6.13)
**IGF-1**	11.10 (5.91, 19.87)
**IGFBP-3**	5.82 (2.78, 11.78)
**IFN-γ**	1.07 (0.24, 4.64)
**IL-10**	4.85 (2.23, 10.22)
**IL-6**	2.33 (0.85, 6.22)
**IL-8**	3.65 (1.56, 8.27)
**TNF-α**	15.72 (8.77, 26.58)
**Insulin**	0.59 (0.06, 5.40)
**Leptin**	1.94 (0.65, 5.62)
**Adiponectin**	7.84 (3.96, 14.93)
**CRP**	0.56 (0.05, 5.56)
**C-Peptide**	0.31 (0.01, 12.58)

ICC: Intra-class correlation coefficient. CI: Confidence interval. DHEA: Dehydroepiandrosterone. SHBG: Sex hormone binding globulin. IGF-1: Insulin-like growth factor-1. IGFBP-3: Insulin-like growth factor binding protein-3. IFN-γ: Interferon gamma. IL-10: Interleukin-10. IL-6: Interleukin-6. IL-8: Interleukin-8. TNF-α: Tumor necrosis factor-alpha. CRP: C-reactive protein. C-peptide: Connecting peptide.

All estimated residual ICCs were below 5% except for IGFBP-3 (6%), adiponectin (8%), IGF-1 (11%), and TNF-α (16%) (Online Resource Table 6.1).

Online Resource 7: Identification, measurement and modelling of sociodemographic and lifestyle confounders

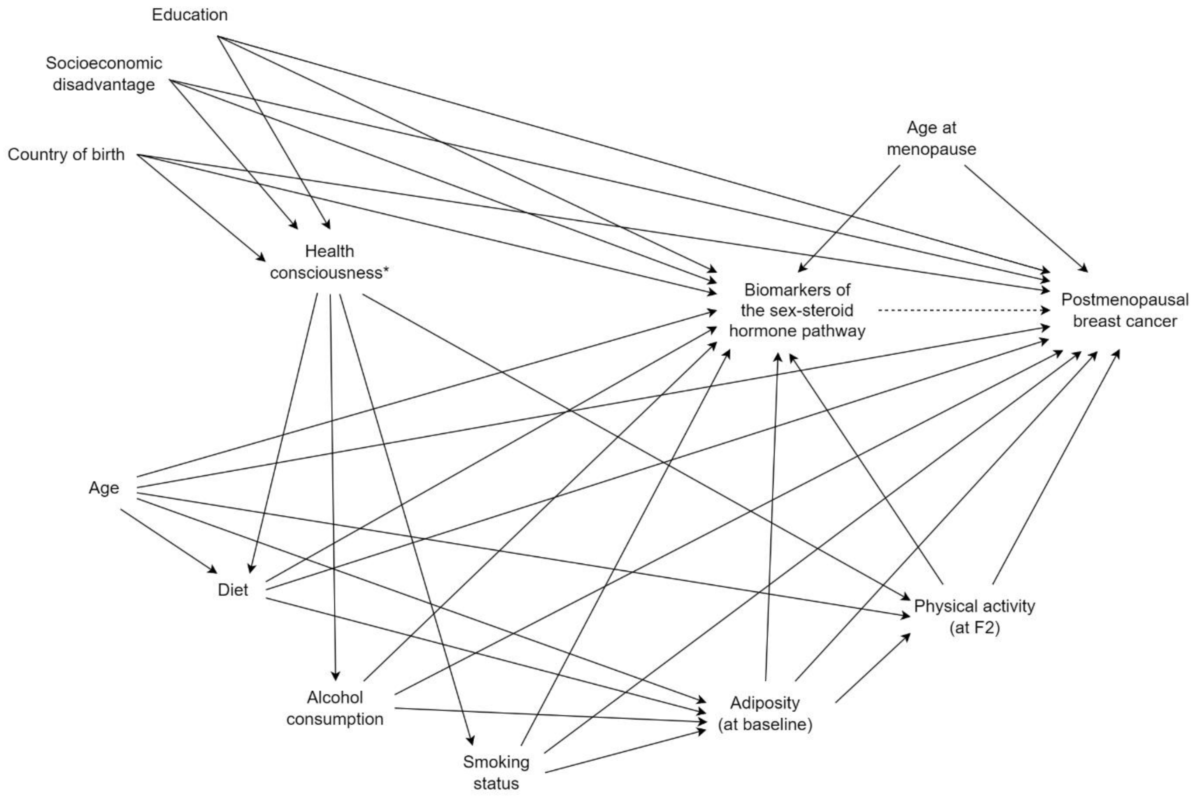

Online Resource Fig. 7.1 Causal diagram of the relationship between biomarkers of the sex-steroid hormone pathway and postmenopausal breast cancer

The causal diagram considers sociodemographic and lifestyle covariates only. Biomarkers of the sex-steroid hormone pathway and physical activity were measured at the second follow-up wave (F2). The measure for age was age at blood collection. Age at menopause was measured when the cessation of periods for 12 months was first reported (baseline, the first follow-up wave, or F2). Postmenopausal breast cancer was measured after F2. All other covariates were measured at baseline. Health consciousness was not measured (indicated by *), but is assumed to influence lifestyle factors (i.e., diet, alcohol consumption, smoking status, physical activity) and be influenced by sociodemographic factors (i.e., country of birth, socioeconomic disadvantage, education).

Highest level of attained education was self-reported at baseline in the Melbourne Collaborative Cohort Study (MCCS) and analyzed as a categorical variable. Country of birth was recorded at baseline and modelled as Southern European migrant status (yes or no). Socioeconomic disadvantage was measured using the Index of Relative Socioeconomic Disadvantage from the Socioeconomic Indexes for Areas (SEIFA) and categorized into

quintiles (of the most to the least disadvantaged, modelled as a continuous variable in the analyses), which were derived at baseline from residential addresses and Australian census data [6].

Dietary factors associated with postmenopausal breast cancer include dietary intake of calcium and dietary intake of carotenoids [7]. Baseline dietary intakes of carotenoids (continuous mcg/d) and dietary calcium intake (continuous mg/d) were derived from a 144-item food frequency questionnaire [6]. Dietary intake of carotenoids was the summation of total dietary intake of alpha-carotene, beta-carotene, beta-cryptoxanthin, lutein and zeaxanthin, and lycopene.

Lifetime alcohol consumption to baseline (continuous g/d) was calculated using self-reported consumption of alcoholic beverages. Baseline smoking status was modelled as a binary variable (ever or never smoked).

Physical activity can arguably influence adiposity, but due to the temporal order of measured variables, adiposity at baseline was assumed to influence physical activity at F2. Adiposity was marked by continuous BMI (kg/m^2) at baseline, derived from height and mass measured at the study center. Physical activity data at F2 were used because the assessment at F2 was more comprehensive than that at baseline. At F2, the duration and frequency of recreation- and transport-related walking, moderate-intensity and vigorous-intensity physical activity of 10 minutes duration or longer across three months was self-reported using a physical activity questionnaire based on the short form of the International Questionnaire of Physical Activity (IPAQ-short) [8]. Physical activity was organized into total weighted minutes of moderate-vigorous physical activity (MVPA) and categorized according to the Physical Activity and Exercise Guidelines for Australians [9]: insufficiently active (< 150 total weighted minutes of MVPA per week); sufficiently active (150 to ≤ 300 total weighted minutes of MVPA per week); and highly active (> 300 total weighted minutes of MVPA per week).

Age was modelled as continuous age at blood collection (years) using restricted cubic splines with three degrees of freedom. Where possible, age at menopause (≤ 48; 49-50; 51-52; ≥ 53 years) was estimated from the data corresponding to when the cessation of periods for 12 months was first reported (baseline, the first follow-up wave, or F2).

Online Resource 8: Identification and selection of biomarker confounders

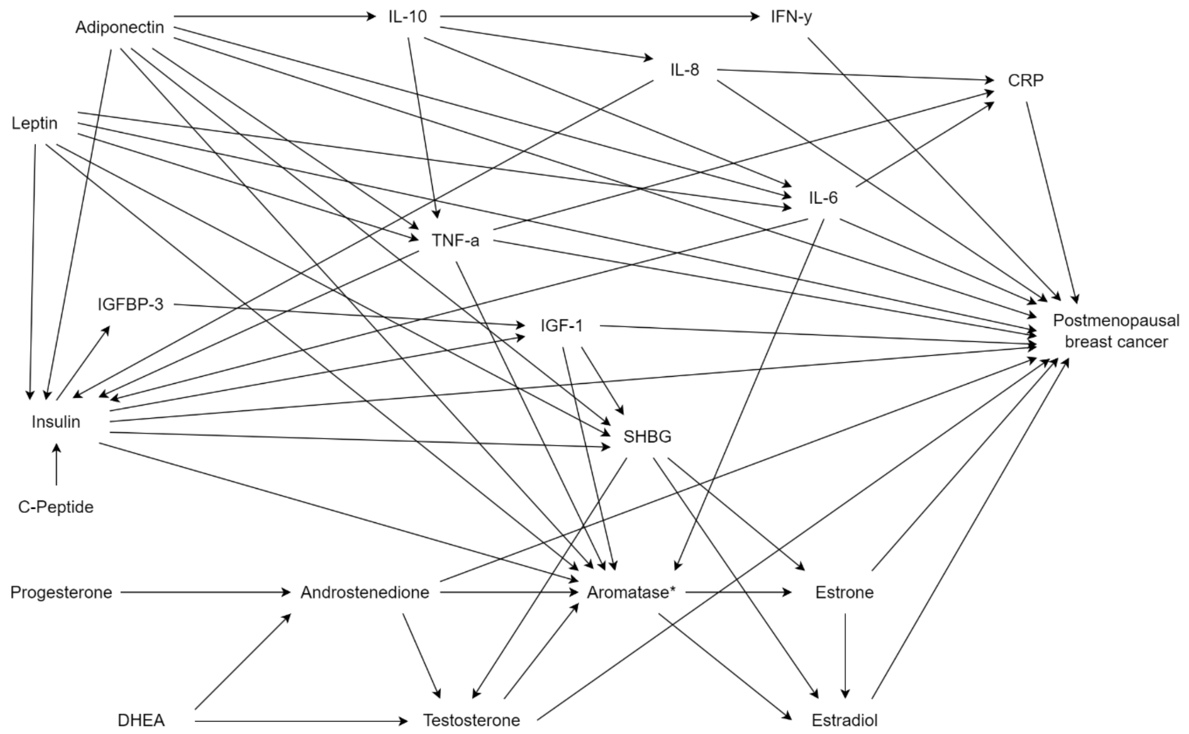

Online Resource Fig. 8.1 Causal diagram of the relationships between measured biomarkers and postmenopausal breast cancer

IL-10: Interleukin-10. IL-8: Interleukin-8. IL-6: Interleukin-6. CRP: C-reactive protein. TNF-a: Tumor necrosis factor-alpha. IFN-y: Interferon gamma. IGFBP-3: Insulin-like growth factor binding protein-3. IGF-1: Insulin-like growth factor-1. SHBG: Sex hormone binding globulin. DHEA: Dehydroepiandrosterone.

Aromatase was not measured (indicated by *) but is depicted as a critical enzyme in steroidogenesis that is influenced by other biomarkers. Otherwise, the causal diagram considers only measured biomarkers as covariates. Arrows depict the assumed net direction of the effects of the biomarkers in this case-cohort of relatively older postmenopausal women.

Biomarkers that may be potential confounders were identified *a priori* using a causal diagram (Online Resource Fig. 8.1) and included in the adjustment set of the primary analysis if they had correlations < 0.50 with the biomarker of interest (Online Resource Table 8.1).

Online Resource Fig. 8.1 was informed by literature review and expert consultation. It was not possible to account for bidirectional relationships and feedback loops as the biomarkers were only measured at one point in time. In general, the inflammatory pathway was assumed

to be upstream of the insulin/insulin-like growth factor (IGF)-signaling pathway, which was assumed to be upstream of the sex-steroid hormone pathway.

Three biomarkers of the sex-steroid hormone pathway were not assumed to have direct effects on postmenopausal breast cancer (Online Resource Fig. 8.1): sex hormone binding globulin (SHBG); progesterone; and DHEA. The inverse association between SHBG and postmenopausal breast cancer risk observed in the literature is assumed to be driven by the role of this glycoprotein in reducing the bioavailability of estrogens and androgens [10-13]. Drummond et al. [13] found moderate-quality evidence that progesterone was not associated with breast cancer, and thus progesterone was assumed to influence breast carcinogenesis indirectly (e.g., via its role as a precursor in steroidogenesis). There was also evidence to suggest that DHEA was not associated with breast cancer in this review [13].

Online Resource Table 8.1 Selection of biomarkers that may be potential confounders in primary analyses

Biomarker	Adjustment Set Inclusions[a]	Adjustment Set Exclusions[b]
**Progesterone**	N/A	N/A
**DHEA**	N/A	N/A
**Androstenedione**	N/A	DHEA. Strong correlation with androstenedione (r = 0.78).
**Testosterone**[c]	SHBG	Androstenedione. Strong correlation with testosterone (r = 0.59).
**SHBG**	Adiponectin, Leptin, Insulin, IGF-1	N/A.
**Estrone**	Adiponectin, Leptin, TNF-α, IL-6, Insulin, IGF-1, SHBG	Androstenedione. Strong correlation with estrone (r = 0.62).
**Estradiol**[c]	Adiponectin, Leptin, TNF-α, IL-6, Insulin, IGF-1, SHBG	Testosterone, estrone. Strong correlation with testosterone (r = 0.54) and estrone (r = 0.85).

N/A: Not applicable. r: Pearson correlation coefficient. DHEA: Dehydroepiandrosterone. SHBG: Sex hormone binding globulin. IGF-1: Insulin-like growth factor-1. TNF-α: Tumor necrosis factor-alpha. IL-6: Interleukin-6.

[a] Biomarkers identified as potential confounders in the causal diagram (Online Resource Fig. 8.1).

[b] Biomarkers identified as potential confounders in the causal diagram (Online Resource Fig. 8.1), but with correlations ≥ 0.50 with the biomarker of interest.

[c] Analyses of free testosterone and estradiol will not adjust for SHBG.

r ≥ 0.50 was considered a strong correlation.

Online Resource 9: Characteristics of the case-cohort after *post-hoc* exclusions

Online Resource Table 9.1 Characteristics of the case-cohort after *post-hoc* exclusions (N = 1,312 women)

	**Cases** N = 378		**Non-Cases** N = 934	
**Age at Blood Collection** (Years; Median, IQR)	66.0	(60.0, 71.0)	68.0	(61.0, 74.0)
**Dietary Calcium Intake** (mg/d; Median, IQR)	803.5	(626.4, 1051.8)	817.6	(608.4, 1044.3)
**Total Carotenoid Intake from Diet** (mcg/d; Median, IQR)	17990	(13781, 24004)	17228	(13352, 23085)
**Southern European Migrant Status** (N, %)				
No	298	78.8%	732	78.4%
Yes	80	21.2%	202	21.6%
**Socioeconomic Disadvantage** (N, %)				
Quintile 1: Most Disadvantaged	56	14.8%	144	15.4%
Quintile 2	66	17.5%	193	20.7%
Quintile 3	61	16.1%	140	15.0%
Quintile 4	79	20.9%	180	19.3%
Quintile 5: Least Disadvantaged	116	30.7%	276	29.6%
**Education** (N, %)				
Primary School or Some High / Technical School	224	59.3%	611	65.4%
Completed High / Technical School	73	19.3%	137	14.7%
Completed Tertiary Degree / Diploma	81	21.4%	186	19.9%
**Smoking Status** (N, %)				
Never Smoked	273	72.2%	687	73.6%
Ever Smoked	105	27.8%	247	26.4%
**Lifetime Alcohol Consumption** (N, %)				
Life Abstention	156	41.3%	346	37.0%
≤ 19 g/d	198	52.4%	531	56.9%
20 to 29 g/d	15	4.0%	26	2.8%
30 to 39 g/d	6	1.6%	19	2.0%
≥ 40 g/d	3	0.8%	12	1.3%
**Body Mass Index** (N, %)				
Normal (≥ 18.5 to < 25 kg/m^2)	151	39.9%	398	42.6%
Overweight (≥ 25 to < 30 kg/m^2)	123	32.5%	338	36.2%
Obese (≥ 30 kg/m^2)	104	27.5%	198	21.2%
**Physical Activity[a]** (N, %)				
Insufficiently Active	115	33.1%	273	31.1%
Sufficiently Active	82	23.6%	248	28.2%
Highly Active	150	43.2%	358	40.7%
**Age at Menopause[b]** (N, %)				

≤ 48 years	47	21.2%	149	24.9%
49-50 years	57	25.7%	151	25.2%
51-52 years	48	21.6%	140	23.4%
≥ 53 years	70	31.5%	159	26.5%
**Normalized Biomarkers** (Median, IQR)				
**Sex-Steroid Hormone Pathway**				
Progesterone (nmol/L)	0.13	(0.10, 0.19)	0.13	(0.10, 0.17)
Androstenedione (nmol/L)	1.5	(1.2, 2.2)	1.5	(1.1, 2.0)
DHEA (nmol/L)	4.9	(3.1, 7.3)	4.4	(2.9, 6.8)
Estrone (pmol/L)	82.2	(61.1, 115.9)	78.3	(58.5, 107.3)
SHBG (nmol/L)	55.3	(40.6, 78.9)	61.8	(45.6, 82.8)
Total Testosterone (nmol/L)	0.64	(0.45, 0.87)	0.62	(0.43, 0.89)
Total Estradiol (pmol/L)	18.8	(12.7, 29.3)	16.3	(10.9, 25.0)
Free Testosterone (pmol/L)	5.5	(3.8, 8.5)	5.1	(3.6, 7.4)
Free Estradiol (pmol/L)	0.26	(0.15, 0.41)	0.20	(0.13, 0.33)
**Insulin/IGF-Signaling Pathway**				
Insulin (pg/mL)	304.2	(211.2, 433.6)	290.3	(209.1, 441.4)
IGF-1 (nmol/L)	7.8	(6.4, 9.4)	7.9	(6.4, 9.9)
IGFBP-3 (nmol/L)	66.2	(58.0, 75.5)	67.9	(58.6, 76.5)
C-Peptide (ng/mL)	2.6	(2.1, 3.4)	2.6	(2.0, 3.4)
**Inflammatory Pathway**				
Leptin (pg/mL)	17586	(8605, 32049)	14056	(6289, 27582)
Adiponectin (ng/mL)	25095	(19055, 32798)	24886	(18762, 33045)
TNF-α (pg/mL)	2.7	(2.2, 3.3)	2.6	(2.2, 3.2)
IL-6 (pg/mL)	0.74	(0.56, 1.04)	0.73	(0.53, 1.02)
IL-8 (pg/mL)	2.9	(2.2, 3.9)	3.0	(2.2, 4.0)
IL-10 (pg/mL)	0.25	(0.19, 0.34)	0.23	(0.17, 0.32)
IFN-γ (pg/mL)	5.6	(4.0, 8.0)	5.5	(3.8, 8.7)
CRP (ng/mL)	1705	(819, 3169)	1386	(691, 3055)

N: Number. IQR: Interquartile range. DHEA: Dehydroepiandrosterone. SHBG: Sex-hormone binding globulin. IGF: Insulin-like growth factor. IGF-1: Insulin-like growth factor-1. IGFBP-3: Insulin-like growth factor binding protein-3. TNF-α: Tumor necrosis growth factor-alpha. IL-6: Interleukin-6. IL-8: Interleukin-8. IL-10: Interleukin-10. IFN-γ: Interferon gamma. CRP: C-reactive protein. nmol/L: Nanomoles per liter. pmol/L: Picomoles per liter. ng/mL: Nanograms per milliliter. pg/mL: Picograms per milliliter. g/d: Grams per day. mg/d: Milligrams per day. mcg/d: Micrograms per day. kg/m^2: Kilograms per meters squared.

[a] Physical activity was measured as total weighted minutes of walking, moderate- and vigorous-intensity recreation- and transport-related physical activity (MVPA) per week at the second follow-up wave. Insufficiently active was defined as < 150 total weighted minutes of MVPA per week, sufficiently active was defined as 150 to ≤ 300 total weighted minutes of MVPA per week, and highly active was defined as > 300 total weighted minutes of MVPA per week.

[b] Age at menopause was measured for naturally postmenopausal women only, when the cessation of periods for 12 months was first documented (baseline, the first follow-up wave, or the second follow-up wave).

Missing data for normalized biomarkers are as follows: 19 for progesterone; 18 for androsterone; 18 for testosterone; 18 for DHEA; 20 for estrone; 27 for estradiol; 18 for SHBG; 2 for insulin; 2 for IGF-1; 2 for IGFBP-3; 2 for C-peptide; 2 for leptin; 3 for adiponectin; 2 for TNF-α; 2 for IL-6; 2 for IL-8; 2 for IFN-γ; 6 for CRP. Missing data for other covariates include: 1 for socioeconomic disadvantage; 86 for physical activity; 491 for age at menopause (including 55 naturally postmenopausal women).

Southern European Migrant status, socioeconomic disadvantage, education, smoking status, lifetime alcohol consumption, body mass index, dietary calcium intake and total carotenoid intake from diet were measured at baseline. Biomarker concentrations, age at blood collection and physical activity were measured at the second follow-up wave.

Online Resource 10: Sensitivity analyses excluding estrogen receptor-negative/progesterone receptor-positive tumors and tumors of unknown hormone receptor status

Online Resource Table 10.1 Risk ratios for postmenopausal estrogen receptor-positive breast cancer per doubling of biomarker concentration, excluding estrogen receptor-negative/progesterone receptor-positive tumors and tumors of unknown hormone receptor status

**Biomarker** (per doubling concentration)	**Cases**	**Subcohort Non-Cases**	**Risk Ratio**	**95% CI**
**Progesterone (nmol/L)**				
Primary analysis	342	865	1.22	(1.03, 1.44)
Excluding ER-/PR+ or unknown tumours	324	865	1.25	(1.05, 1.48)
**Androstenedione (nmol/L)**				
Primary analysis	342	866	1.20	(0.99, 1.45)
Excluding ER-/PR+ or unknown tumours	324	866	1.24	(1.02, 1.51)
**DHEA (nmol/L)**				
Primary analysis	342	866	1.15	(1.00, 1.34)
Excluding ER-/PR+ or unknown tumours	324	866	1.19	(1.03, 1.38)
**Total Testosterone (nmol/L)**				
Primary analysis (adjusted for SHBG)	342	866	1.11	(0.96, 1.29)
Excluding ER-/PR+ or unknown tumours	324	866	1.13	(0.97, 1.31)
**Free Testosterone (nmol/L)**				
Primary analysis	342	866	1.12	(0.98, 1.28)
Excluding ER-/PR+ or unknown tumours	324	866	1.14	(1.00, 1.31)
**Estrone (pmol/L)**				
Primary analysis (adjusted for adiponectin, leptin, TNF-α, IL-6, insulin, IGF-1 and SHBG)	342	863	1.21	(0.99, 1.48)
Excluding ER-/PR+ or unknown tumours	324	863	1.23	(1.00, 1.52)
**Total Estradiol (pmol/L)**				
Primary analysis (adjusted for adiponectin, leptin, TNF-α, IL-6, insulin, IGF-1 and SHBG)	341	858	1.19	(1.02, 1.39)
Excluding ER-/PR+ or unknown tumours	323	858	1.21	(1.03, 1.41)
**Free Estradiol (pmol/L)**				
Primary analysis (adjusted for adiponectin, leptin, TNF-α, IL-6, insulin and IGF-1)	341	858	1.22	(1.05, 1.41)
Excluding ER-/PR+ or unknown tumours	323	858	1.24	(1.07, 1.44)
**SHBG (nmol/L)**				
Primary analysis (adjusted for adiponectin, leptin, insulin and IGF-1)	342	865	0.83	(0.66, 1.05)
Excluding ER-/PR+ or unknown tumours	324	865	0.80	(0.63, 1.02)

CI: Confidence interval. ER-: Estrogen receptor-negative. PR+: Progesterone receptor-positive. DHEA: Dehydroepiandrosterone. SHBG: Sex hormone binding globulin. IGF-1: Insulin-like growth factor-1. IL-6:

Interleukin-6. TNF-α: Tumor necrosis factor-alpha. nmol/L: Nanomoles per liter. pmol/L: Picomoles per liter.

The results of the primary analyses and sensitivity analyses were adjusted for sociodemographic and lifestyle confounders (education, socioeconomic disadvantage, Southern European Migrant status, dietary intake of carotenoids at baseline, dietary intake of calcium at baseline, lifestyle alcohol consumption at baseline, smoking status at baseline, adiposity at baseline, physical activity at the second follow-up wave and age at blood collection) and other biomarkers identified as potential confounders, where applicable (Online Resource 8). The sensitivity analyses additionally excluded cases that were ER-/PR+ or of unknown hormone receptor status.

Online Resource 11: Sensitivity analyses excluding cases and deaths that occurred within one year of blood draw at the second follow-up wave

Online Resource Table 11.1 Risk ratios for postmenopausal estrogen receptor-positive breast cancer per doubling of biomarker concentration, excluding cases and deaths that occurred within one year of blood draw at the second follow-up wave

**Biomarker** (per doubling concentration)	**Cases**	**Subcohort Non-Cases**	**Risk Ratio**	**95% CI**
**Progesterone (nmol/L)**				
Primary analysis	342	865	1.22	(1.03, 1.44)
Excluding cases and deaths < 1 year from F2	322	861	1.22	(1.03, 1.45)
**Androstenedione (nmol/L)**				
Primary analysis	342	866	1.20	(0.99, 1.45)
Excluding cases and deaths < 1 year from F2	322	861	1.19	(0.98, 1.45)
**DHEA (nmol/L)**				
Primary analysis	342	866	1.15	(1.00, 1.34)
Excluding cases and deaths < 1 year from F2	322	861	1.15	(0.99, 1.34)
**Total Testosterone (nmol/L)**				
Primary analysis (adjusted for SHBG)	342	866	1.11	(0.96, 1.29)
Excluding cases and deaths < 1 year from F2	322	861	1.11	(0.96, 1.29)
**Free Testosterone (nmol/L)**				
Primary analysis	342	866	1.12	(0.98, 1.28)
Excluding cases and deaths < 1 year from F2	322	861	1.11	(0.97, 1.27)
**Estrone (pmol/L)**				
Primary analysis (adjusted for adiponectin, leptin, TNF-α, IL-6, insulin, IGF-1 and SHBG)	342	863	1.21	(0.99, 1.48)
Excluding cases and deaths < 1 year from F2	322	858	1.17	(0.96, 1.44)
**Total Estradiol (pmol/L)**				
Primary analysis (adjusted for adiponectin, leptin, TNF-α, IL-6, insulin, IGF-1 and SHBG)	341	858	1.19	(1.02, 1.39)
Excluding cases and deaths < 1 year from F2	321	854	1.17	(1.00, 1.37)
**Free Estradiol (pmol/L)**				
Primary analysis (adjusted for adiponectin, leptin, TNF-α, IL-6, insulin and IGF-1)	341	858	1.22	(1.05, 1.41)
Excluding cases and deaths < 1 year from F2	321	854	1.19	(1.02, 1.38)
**SHBG (nmol/L)**				
Primary analysis (adjusted for adiponectin, leptin, insulin and IGF-1)	342	865	0.83	(0.66, 1.05)
Excluding cases and deaths < 1 year from F2	322	860	0.85	(0.67, 1.09)

CI: Confidence interval. F2: Follow-up wave two. DHEA: Dehydroepiandrosterone. SHBG: Sex hormone binding globulin. IGF-1: Insulin-like growth factor-1. IL-6: Interleukin-6. TNF-α: Tumor necrosis factor-alpha. nmol/L: Nanomoles per liter. pmol/L: Picomoles per liter.

The results of the primary analyses and sensitivity analyses were adjusted for sociodemographic and lifestyle

confounders (education, socioeconomic disadvantage, Southern European Migrant status, dietary intake of carotenoids at baseline, dietary intake of calcium at baseline, lifestyle alcohol consumption at baseline, smoking status at baseline, adiposity at baseline, physical activity at the second follow-up wave and age at blood collection) and other biomarkers identified as potential confounders, where applicable (Online Resource 8). The sensitivity analyses additionally excluded cases and deaths that occurred within one year of blood draw at F2.

Online Resource 12: Sensitivity analyses for naturally postmenopausal women with a recorded age at menopause

Online Resource Table 12.1 Risk ratios for postmenopausal estrogen receptor-positive breast cancer per doubling of biomarker concentration, for naturally postmenopausal women with a recorded age at menopause

**Biomarker** (per doubling concentration)	**Cases**	**Subcohort Non-Cases**	**Risk Ratio**	**95% CI**
**Progesterone (nmol/L)**				
Adjusted for age at menopause	197	559	1.11	(0.90, 1.36)
Not adjusted for age at menopause	197	559	1.13	(0.92, 1.38)
**Androstenedione (nmol/L)**				
Adjusted for age at menopause	197	559	1.08	(0.85, 1.39)
Not adjusted for age at menopause	197	559	1.09	(0.86, 1.40)
**DHEA (nmol/L)**				
Adjusted for age at menopause	197	559	1.15	(0.95, 1.38)
Not adjusted for age at menopause	197	559	1.15	(0.95, 1.39)
**Total Testosterone (nmol/L)**				
Adjusted for age at menopause and SHBG	197	559	1.10	(0.91, 1.33)
Not adjusted for age at menopause	197	559	1.10	(0.91, 1.33)
Not adjusted for other biomarkers	197	559	1.09	(0.90, 1.32)
Not adjusted for other biomarkers and age at menopause	197	559	1.09	(0.90, 1.32)
**Free Testosterone (nmol/L)**				
Adjusted for age at menopause	197	559	1.11	(0.94, 1.32)
Not adjusted for age at menopause	197	559	1.11	(0.94, 1.32)
**Estrone (pmol/L)**				
Adjusted for age at menopause, adiponectin, leptin, TNF-α, IL-6, insulin, IGF-1 and SHBG	197	556	1.30	(0.99, 1.69)
Not adjusted for age at menopause	197	556	1.31	(1.00, 1.70)
Not adjusted for other biomarkers	197	557	1.24	(0.97, 1.59)
Not adjusted for other biomarkers and age at menopause	197	557	1.25	(0.98, 1.60)
**Total Estradiol (pmol/L)**				
Adjusted for age at menopause, adiponectin, leptin, TNF-α, IL-6, insulin, IGF-1 and SHBG	197	553	1.29	(1.04, 1.58)
Not adjusted for age at menopause	197	553	1.28	(1.04, 1.58)
Not adjusted for other biomarkers	197	554	1.27	(1.05, 1.53)
Not adjusted for other biomarkers and age at menopause	197	554	1.27	(1.05, 1.53)
**Free Estradiol (pmol/L)**				
Adjusted for age at menopause, adiponectin, leptin, TNF-α, IL-6, insulin and IGF-1	197	553	1.31	(1.08, 1.60)

Not adjusted for age at menopause	197	553	1.31	(1.08, 1.59)
Not adjusted for other biomarkers	197	554	1.23	(1.04, 1.46)
Not adjusted for other biomarkers and age at menopause	197	554	1.24	(1.04, 1.47)
**SHBG (nmol/L)**				
Adjusted for age at menopause, adiponectin, leptin, insulin and IGF-1	197	558	0.82	(0.61, 1.09)
Not adjusted for age at menopause	197	558	0.82	(0.61, 1.09)
Not adjusted for other biomarkers	197	559	0.89	(0.68, 1.16)
Not adjusted for other biomarkers and age at menopause	197	559	0.88	(0.68, 1.15)

CI: Confidence interval. DHEA: Dehydroepiandrosterone. SHBG: Sex hormone binding globulin. IGF-1: Insulin-like growth factor-1. IL-6: Interleukin-6. TNF-α: Tumor necrosis factor-alpha. nmol/L: Nanomoles per liter. pmol/L: Picomoles per liter.

Results were adjusted for sociodemographic and lifestyle confounders (education, socioeconomic disadvantage, Southern European Migrant status, dietary intake of carotenoids at baseline, dietary intake of calcium at baseline, lifestyle alcohol consumption at baseline, smoking status at baseline, adiposity at baseline, physical activity at the second follow-up wave and age at blood collection). Results were also adjusted for age at menopause and other biomarkers identified as potential confounders (where applicable, Online Resource 8), unless otherwise specified.

Online Resource 13: Quartile minimum, median and maximum values

Online Resource Table 13.1 Minimum, median and maximum values of the quartiles of normalized biomarker concentrations

Biomarker	Quartile[a]	Minimum	Median	Maximum
**Progesterone (nmol/L)**	1	0.02	0.08	0.10
	2	0.10	0.11	0.13
	3	0.13	0.15	0.17
	4	0.17	0.22	3.18
**Androstenedione (nmol/L)**	1	0.17	0.90	1.15
	2	1.15	1.36	1.54
	3	1.54	1.75	2.01
	4	2.01	2.50	10.09
**DHEA (nmol/L)**	1	0.21	2.06	2.85
	2	2.85	3.64	4.39
	3	4.40	5.59	6.82
	4	6.82	8.84	21.87
**Total Testosterone (nmol/L)**	1	0.01	0.35	0.43
	2	0.43	0.52	0.62
	3	0.62	0.74	0.89
	4	0.89	1.15	21.85
**Free Testosterone (pmol/L)**	1	0.16	2.53	3.56
	2	3.57	4.28	5.11
	3	5.11	6.06	7.50
	4	7.51	10.09	263.69
**Estrone (pmol/L)**	1	2.74	47.59	58.16
	2	58.30	67.14	78.05
	3	78.09	90.64	107.25
	4	107.25	133.47	562.33
**Total Estradiol (pmol/L)**	1	1.98	7.98	10.85
	2	10.86	13.55	16.21
	3	16.22	20.12	25.06
	4	25.13	34.29	108.14
**Free Estradiol (pmol/L)**	1	0.01	0.09	0.13
	2	0.13	0.16	0.20
	3	0.20	0.26	0.33
	4	0.33	0.49	2.11
**SHBG (nmol/L)**	1	8.52	35.77	45.42
	2	45.44	53.84	61.44
	3	61.49	71.12	82.81
	4	82.95	103.44	262.69

DHEA: Dehydroepiandrosterone. SHBG: Sex hormone binding globulin. nmol/L: Nanomoles per liter. pmol/L: Picomoles per liter.

[a] Quartiles based on the distribution of normalized biomarker values in the subcohort.

Supplementary References

1. Young JG, Stensrud MJ, Tchetgen Tchetgen EJ, Hernán MA. (2020) A causal framework for classical statistical estimands in failure-time settings with competing events. Statistics in medicine. 39: 1199-236. https://doi.org/10.1002/sim.8471.
2. Hernán MA, Robins JM. (2023) Causal Inference: What If. Boca Raton: Chapman & Hall/CRC.
3. Liljequist D, Elfving B, Skavberg Roaldsen K. (2019) Intraclass correlation - A discussion and demonstration of basic features. PloS one. 14: e0219854. https://doi.org/10.1371/journal.pone.0219854.
4. Ionan AC, Polley M-YC, McShane LM, Dobbin KK. (2014) Comparison of confidence interval methods for an intra-class correlation coefficient (ICC). BMC medical research methodology. 14: 121. https://doi.org/10.1186/1471-2288-14-121.
5. Viallon V, His M, Rinaldi S, et al. (2021) A new pipeline for the normalization and pooling of metabolomics data. Metabolites. 11: 631. https://doi.org/doi:10.3390/metabo11090631.
6. Milne RL, Fletcher AS, MacInnis RJ, et al. (2017) Cohort profile: The Melbourne Collaborative Cohort Study (Health 2020). Int J Epidemiol. 46: 1757-i. https://doi.org/10.1093/ije/dyx085.
7. (2018) Continuous Update Project Expert Report 2018. Diet, Nutrition, Physical Activity and Breast Cancer. World Cancer Research Fund / American Institute for Cancer Research.
8. (2005) Guidelines for Data Processing and Analysis of the International Physical Activity Questionnaire (IPAQ) - Short and Long Forms. IPAQ Group.
9. Kirkwood BR, Sterne, J. AC. (2003) Essential Medical Statistics. 2nd ed: Wiley-Blackwell.
10. Friedenreich CM, Ryder-Burbidge C, McNeil J. (2021) Physical activity, obesity and sedentary behavior in cancer etiology: epidemiologic evidence and biologic mechanisms. Mol Oncol. 15: 790-800. https://doi.org/10.1002/1878-0261.12772.
11. Lynch BM, Leitzmann MF. (2017) An evaluation of the evidence relating to physical inactivity, sedentary behavior, and cancer incidence and mortality. Current Epidemiology Reports. 4: 221-31. https://doi.org/10.1007/s40471-017-0119-7.
12. Simó R, Sáez-López C, Barbosa-Desongles A, Hernández C, Selva DM. (2015) Novel insights in SHBG regulation and clinical implications. Trends in endocrinology and metabolism: TEM. 26: 376-83. https://doi.org/10.1016/j.tem.2015.05.001.

13. Drummond AE, Swain CTV, Brown KA, et al. (2022) Linking physical activity to breast cancer via sex steroid hormones, part 2: The effect of sex steroid hormones on breast cancer risk. Cancer Epidemiol Biomarkers Prev. 31: 28-37. https://doi.org/10.1158/1055-9965.Epi-21-0438.
